# Supplementary material for: Eimeria maxima Rhomboid-like Protein 5 Provided Partial Protection against Homologous Challenge in Forms of Recombinant Protein and DNA Plasmid in Chickens
Source: Vaccines (Basel). 2021 Dec 27;10(1):32. doi: 10.3390/vaccines10010032 (PMC8781051; doi:10.3390/vaccines10010032)
Supplement: Supplementary file 1 [file vaccines-10-00032-s001.zip › vaccines-1476624-supplementary.pdf]

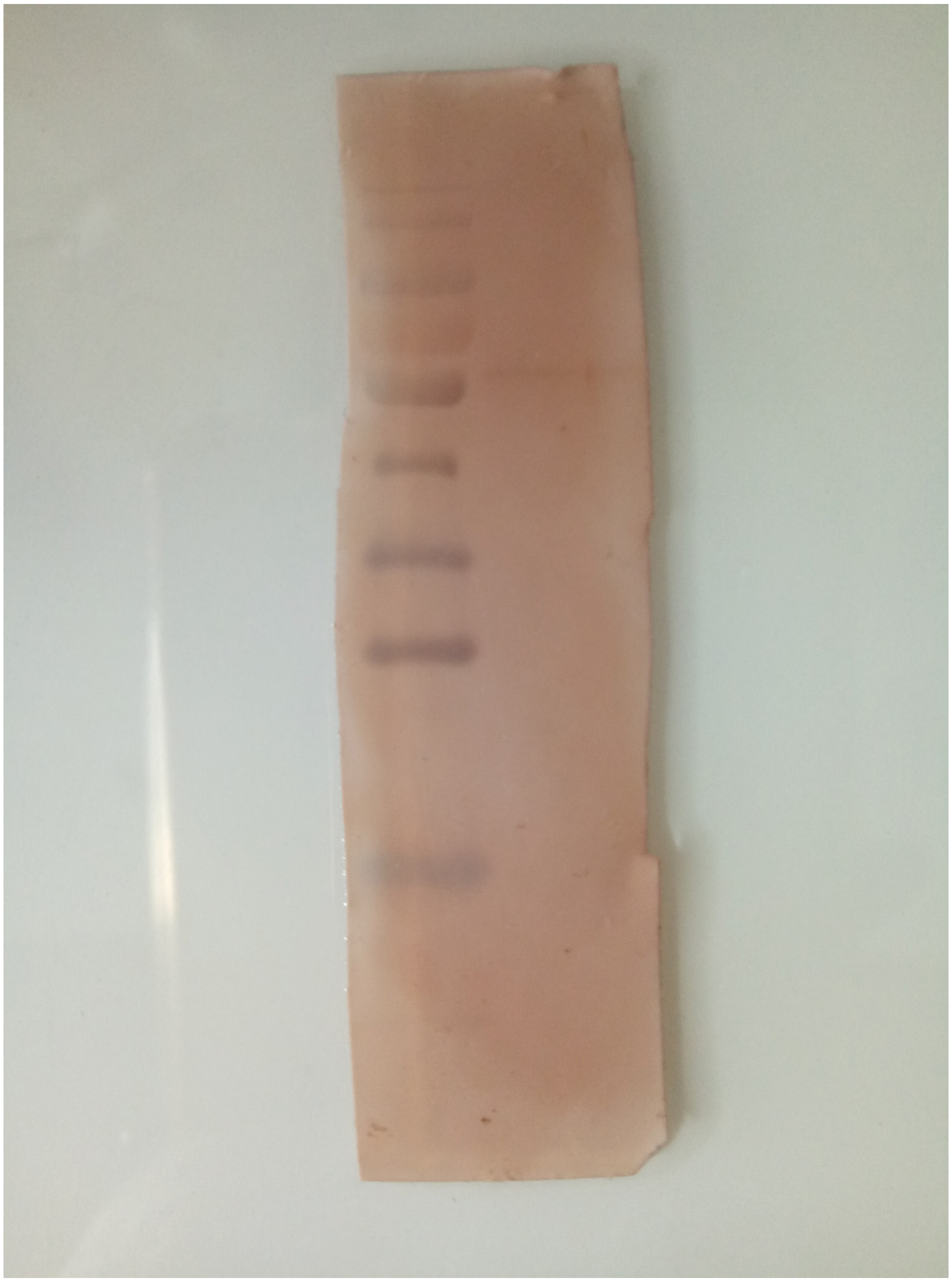

Figure S1. The original image for figure 2C lane 1 in the main article.

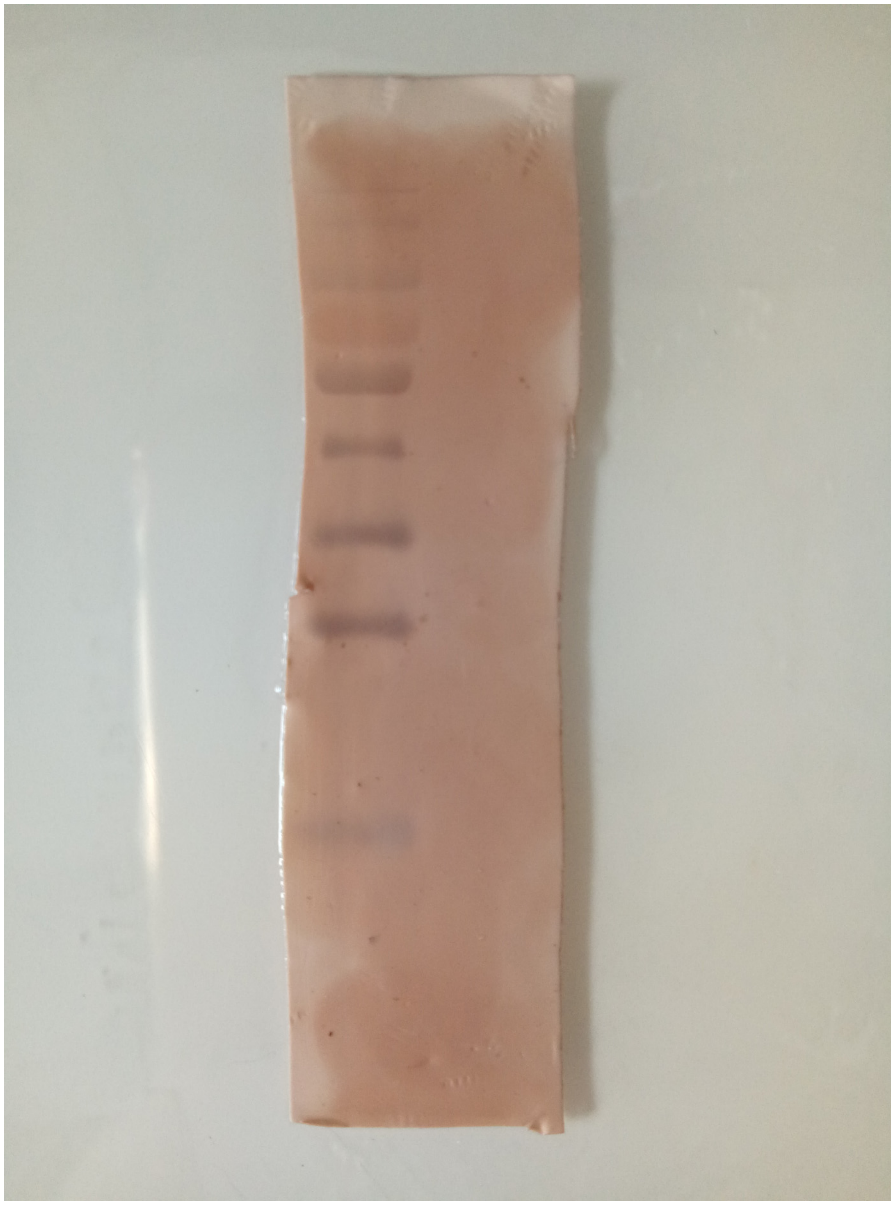

Figure S2. The original image for figure 2C lane 2 in the main article.

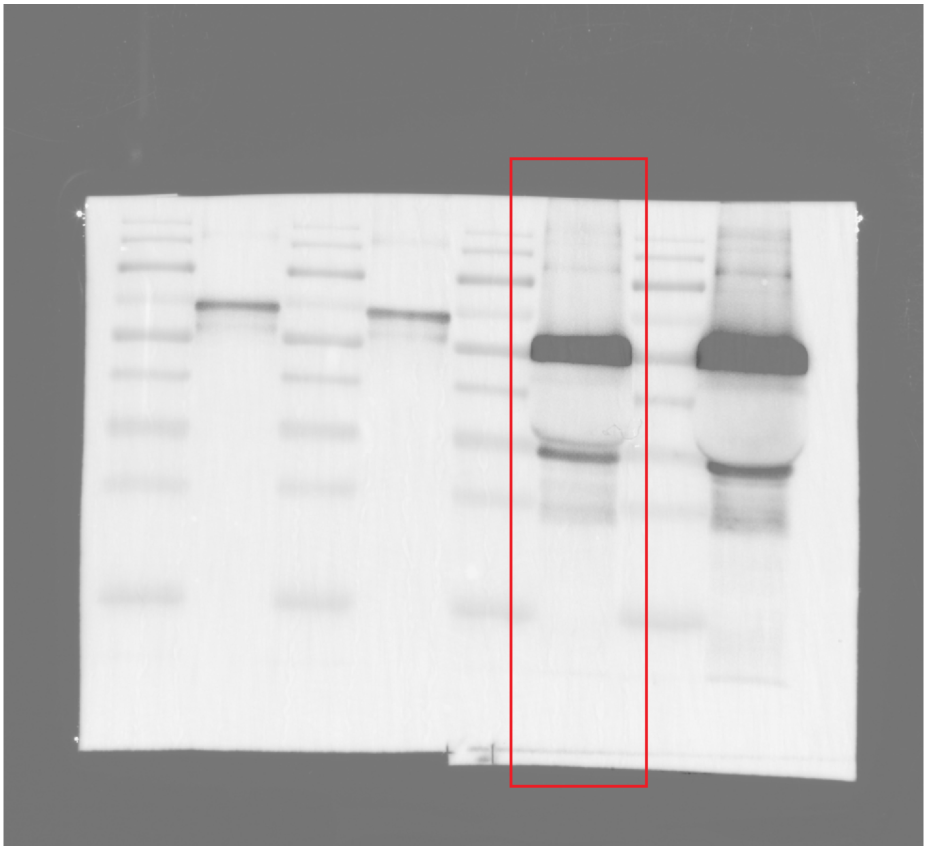

Figure S3. The original image for figure 2C lane 3 in the main article.

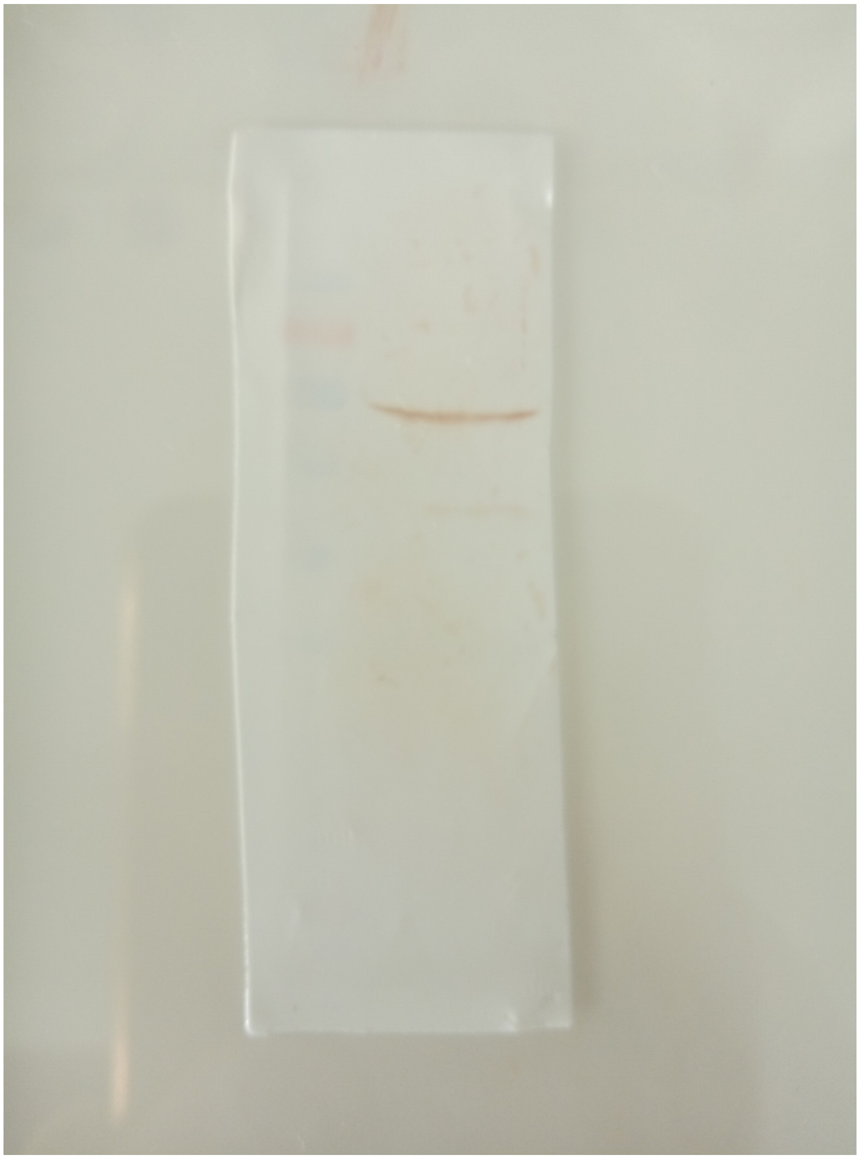

Figure S4. The original image for figure 3B lane 1 in the main article.

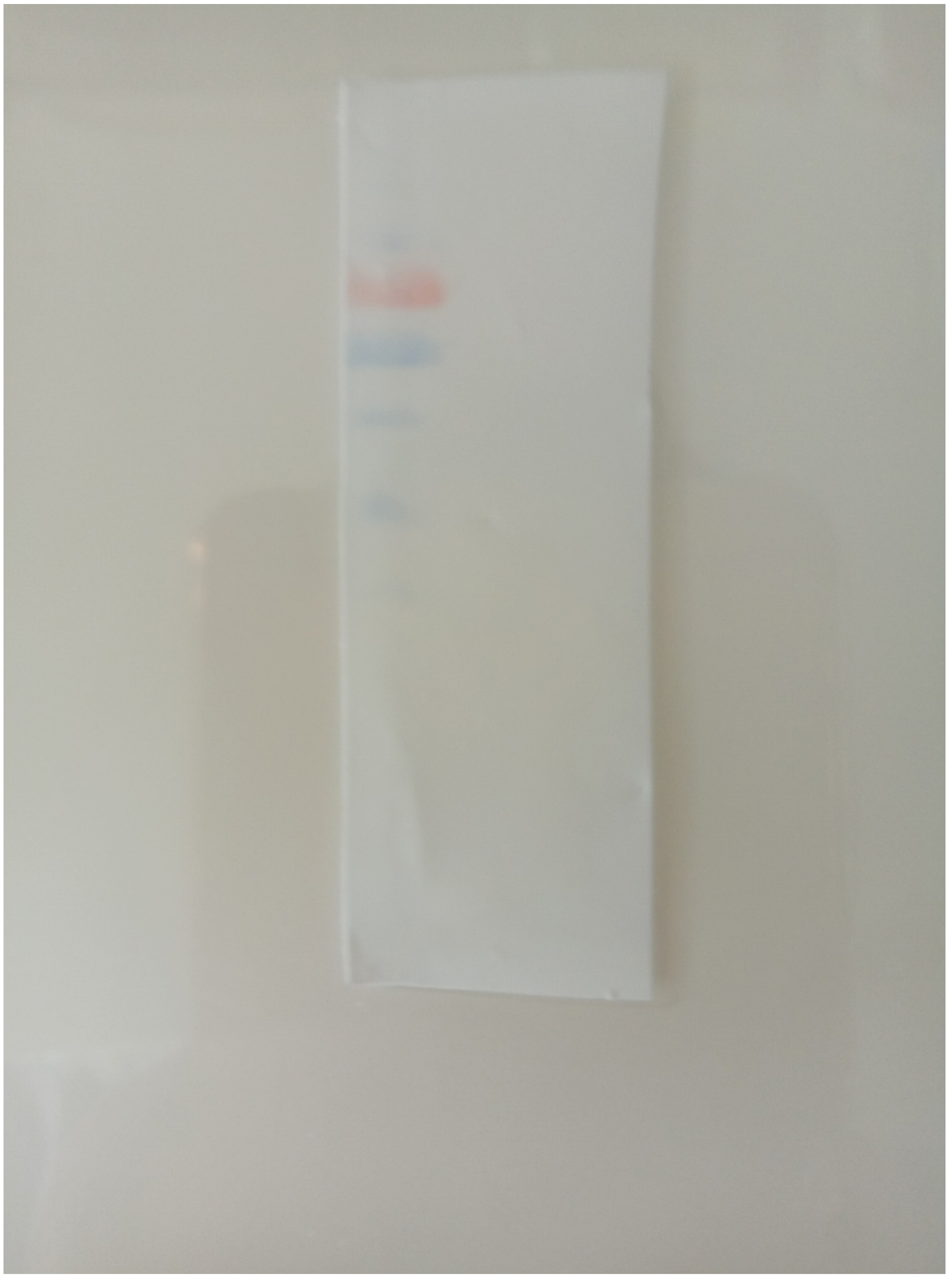

Figure S5. The original image for figure 3B lane 2 in the main article.
